# MIST1 regulates SNAI1 and acts through the PTEN/AKT signaling axis to promote anoikis resistance in human melanoma cells

**DOI:** 10.3892/etm.2020.9481

**Published:** 2020-11-17

**Authors:** Yiju Lee, Weifeng Yao, Chunjun Yang, Yunrui Li, Haiyang Ni, Lei Wang, Bin Ji, Yongge Gu, Sen Yang

Exp Ther Med 16:695–703, 2018; DOI: 10.3892/etm.2018.6225

After the publication of the above paper, the first author (Yiju Lee) has realized that their name was associated with the incorrect affiliation; instead of being listed as a member of the Graduate School (Anhui Medical University, Hefei, Anhui), Yiju Lee’s affiliation should have been given as “Department of Dermatology, The First Affiliated Hospital of Anhui Medical University, Hefei, Anhui 230022, P.R. China”, as per the two PhD tutors (Chunjun Yang and Sen Yang) involved. Therefore, the authors’ names and affiliations in this paper should have been presented as follows:

YIJU LEE^1*^, WEIFENG YAO^2*^, CHUNJUN YANG^1^, YUNRUI LI^3^, HAIYANG NI^2^, LEI WANG^2^, BIN JI^2^, YONGGE GU^2^ AND SEN YANG^1^

^1^Department of Dermatology, The First Affiliated Hospital of Anhui Medical University, Hefei, Anhui 230022; ^2^Department of Dermatology, Tianjin Academy of Traditional Chinese Medicine Affiliated Hospital, Tianjin 300120; ^3^School of Basic Medicine, Tianjin Medical University, Tianjin 300070, P.R. China

^*^Contributed equally

The authors regret that this error with the author affiliations was not noticed prior to the publication of their paper, and apologize for any inconvenience caused.

